# The impact of COVID‐19 on adolescent wellness in Chicago

**DOI:** 10.1111/cch.12994

**Published:** 2022-03-10

**Authors:** Ogochukwu M. Ezeoke, Madeleine K. Kanaley, Dannielle A. Brown, Olivia R. Negris, Rajeshree Das, Lisa S. Lombard, Ruchi S. Gupta

**Affiliations:** ^1^ Ann & Robert H. Lurie Children's Hospital of Chicago Chicago Illinois USA; ^2^ Institute for Public Health and Medicine, Center for Food Allergy and Asthma Research Northwestern University Feinberg School of Medicine Chicago Illinois USA

**Keywords:** adolescence, development, social relationships, social support, stress, well‐being

## Abstract

**Purpose:**

This study aimed to understand the impact of the initial COVID‐19 pandemic remote schooling period on self‐reported wellness among adolescents in Chicago.

**Methods:**

Students (*n* = 55) completed a 22‐item wellness questionnaire before (February 2020) and shortly after the onset of the COVID‐19 outbreak (April 2020). Precomparisons/postcomparisons (overall and by survey item) were evaluated using two‐sided paired t‐tests with an alpha level of 0.05. Descriptive statistics were used to evaluate mean scores overall by demographic variables.

**Results:**

Significant differences were found in the following areas: Balance (Pre: 7.3, During: 6.4, *p* = 0.02), Education (Pre: 8.4, During 7.7, *p* = 0.03) and Friends (Pre:8.0, During: 6.3, *p* = 0.001). Overall wellness scores varied by demographic variables, though not significantly.

**Conclusions:**

Results suggest the onset of the pandemic impacted students' ability to effectively learn, as well as to maintain balance in their lives and social relationships. Comprehensive support is needed in these areas to promote adolescent wellness.

**Key messages**

- Adolescent wellness is integral to healthy psychosocial development into young adulthood.
- There is limited data available for clinical measurement of wellness in adolescents, from a holistic perspective (e.g., when not including primary depression assessments).
- Our study aimed to assess wellness in adolescents through a comprehensive study that addressed 22 points of interest associated with overall wellness.
- Our study demonstrated that COVID‐19 pandemic may be significantly affecting adolescent wellness in three specific ways: social relationships, education, and lifestyle balance.
- Research targeted at alleviating the burden on adolescents during the ongoing pandemic should consider these factors.

## 1 INTRODUCTION

Adolescence is a challenging time of biological development and psychosocial maturation (Rowley et al., 2005), for which wellness plays a critical role. The World Health Organization (WHO) defines wellness as “the optimal state of health of individuals and groups” with a focus on realizing one's fullest potential and fulfilling one's role expectations (Smith et al., 2006). Understanding adolescent wellness using a holistic approach has been an emerging priority among health experts (Spurr et al., 2012), and there is increased urgency for this given the wide‐ranging impacts of COVID‐19 on adolescent behavioural health (United States Public Health Service, Office of the Surgeon General, 2021). While some plans exist to address this gap by creating more comprehensive assessments of the physical, psychological, spiritual, and social aspects of adolescents' lives, these approaches are not utilized in routine adolescent clinical practice (Bart et al., 2018; Hattie et al., 2004; Katja et al., 2002; Myers et al., 2000; Spurr et al., 2012; Steiner et al., 1998). The initial phase of the pandemic and the concomitant shift to remote schooling and social distancing magnify how important the holistic perspective is for understanding adolescent wellness.

Social distancing measures due to the pandemic disrupted daily routines (Lee, 2020) and left many students socially isolated without access to school‐based coping resources that provided support and helped them maintain a healthy lifestyle. Previous research has shown longitudinal evidence for the decline in adolescents' mental health during the COVID‐19 pandemic. Using an online survey, adolescent life satisfaction and depressive and anxious symptoms before the pandemic restrictions and 2 months after remote learning and lockdown were studied. Findings indicated that adolescents experienced significant increases in depressive symptoms and anxiety, and a significant decrease in life satisfaction (Cohen et al., 2021). Feeling socially connected protected against poor mental health, while online learning difficulties and conflict with parents predicted increases in mental health problems across time.

To understand the short‐term and potential long‐term effects of the pandemic on adolescent well‐being, changes in self‐reported wellness factors were explored. These findings can inform paediatricians and other adolescent health practitioners about areas to consider when identifying behavioural health needs and developing tools for effective care. This study aimed to examine adolescent wellness from a holistic perspective so that a fuller understanding of their experiences during the onset of the COVID‐19 pandemic could inform future clinical care.

## 2 METHODS

Ninety‐five students from 16 public high schools in Chicago participated in a programme and research study designed to help students become health leaders by creating peer‐facing educational materials on community health issues. They were recruited to participate through an invitational flier sent to district principals and science teachers. Exclusion criteria included students over 18 years old or parents unable to provide consent. Institutional Review Board approval was obtained for this research.

A 22‐item survey evaluating adolescent wellness was developed by physicians and researchers at the primary institution's associated medical school, and the affiliated urban children's hospital, to explore students' academic, social, physical, and creative ability, as well as factors such as balance, growth mindset, friends, and goals. Development of the survey involved a focus group of 18 adolescents and young adults to discuss the concept of wellness and create a list of terms associated with wellness. Following this meeting, a prototype survey was developed containing 31 questions. This survey was disseminated amongst the original focus group for readability, and to assess interpretation of the survey questions. After initial survey completion, members of the research team assessed the answers for concordance of interpretation with the intent of the questions, and to screen questions for redundancy.

The survey was disseminated to students prior to the COVID‐19 outbreak (February 2020), and once during the pandemic (April 2020). Students rated items on a scale of 1 (*hardly ever*) to 10 (*all of the time*) “in the past week.” Descriptive statistics were used to evaluate overall mean scores by demographic variables such as grade in school, race/ethnicity, employment status, and education. Additionally, precomparisons/postcomparisons in overall scores and individual survey items were evaluated using two‐sided paired *t*‐tests with an alpha level of 0.05. All statistical analyses were performed using Python (Rossum & Drake, 2009).

## 3 RESULTS

**FIGURE 1:**
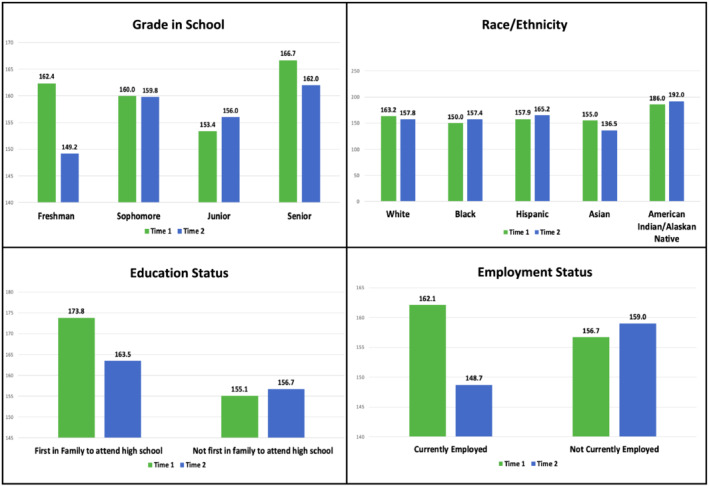
Average overall wellness scores pre and during COVID‐19

Of the 95 students enrolled, 55 students completed the survey at both time points. The majority identified as female (81.8%). Many were Sophomores (40.0%) or Juniors (45.5%) in high school and identified as Hispanic (43.6%), Black (20.0%), Asian (18.2%), and White (18.2%). When evaluating scores per item, students had statistically significant differences in scores on the following items (Table 1): Balance (*Felt like you devoted appropriate amounts of time to all the important areas of your life*) (Pre: 7.3, During: 6.4, *p* = 0.02), Education (*Learned something valuable or interesting*) (Pre: 8.4, During 7.7, *p* = 0.03) and Friends (*Spent quality time with friends*) (Pre:8.0, During:6.3, *p* = 0.001). There were no significant differences in overall composite wellness scores (out of 220) (Pre: 157.6, During: 157.2, *p* = 0.96) or when evaluating overall scores by demographic variables (Figure 1). However, there was an observed increase in scores among juniors (Pre: 153.4, During 156.0, *p* = 0.82), Black (Pre: 150.0 During: 157.4, *p* = 0.69) and Hispanic (Pre:157.9, During: 165.2, *p* = 0.52) students, and decrease for freshmen (Pre: 162.4, During 149.2, *p* = 0.45), senior (Pre: 166.7, During: 162.0, *p* = 0.70), White (163.2, 157.8, *p* = 0.63), and Asian (Pre: 155.0, During: 136.5, *p* = 0.14) students who are first in the family to attend high school (Pre: 173.8, During: 163.5, *p* = 0.42) and students who are currently employed (Pre: 162.1, During: 148.7, *p* = 0.25).

**TABLE 1:** Differences in wellness score by survey item

	Pre‐COVID‐19		During COVID‐19		
	Average score	95% CI	Average score	95% CI	*p* value
Wellness survey item (scale of 1–10) (*hardly ever* to *all of the time*)					
Activity: Engage in activities that spark joy	7.9	(6.9, 8.1)	7.4	(6.9, 7.9)	0.08
Balance: Felt like you devoted appropriate amounts of time to all the important areas of your life	7.3	(6.2, 7.6)	6.4	(5.8, 6.9)	0.02
Community: Spend time with your community (whatever community means to you)	7.5	(6.4, 7.8)	6.8	(6.1, 7.4)	0.07
Dreams: Feel excited by what your future may hold	8.4	(7.2, 8.6)	7.6	(7.2, 8.2)	0.09
Education: Learned something valuable or interesting	8.4	(7.3, 8.6)	7.7	(7.1, 8.2)	0.03
Encouragement: Felt supported by those around you	8.1	(7.0, 8.3)	7.5	(6.7, 8.1)	0.09
Expectations: Felt you were meeting your own expectations of yourself	6.9	(5.9, 7.2)	6.6	(6.0, 7.1)	0.36
Expectations: Felt you were meeting other's expectations of you	6.8	(5.7, 7.0)	6.5	(5.9, 7.0)	0.50
Finances: Worried about spending money	6.9	(5.7, 7.3)	5.9	(5.1, 6.7)	0.06
Food: Feel good about the food you eat	7.1	(5.9, 7.5)	7.1	(6.5, 7.7)	0.93
Friends: Spent quality time with friends	8.0	(6.8, 8.2)	6.3	(5.6, 7.0)	0.0001
Family: Spent quality time with family	7.10	(5.9, 7.5)	7.4	(6.9, 8.0)	0.46
Goals: Spent time thinking about accomplishing your goals	8.1	(6.9, 8.3)	7.8	(7.3, 8.3)	0.40
Growth mindset: Felt like the work you have done will help you grow and change for the better	8.3	(7.2, 8.4)	7.7	(7.2, 8.2)	0.07
Growth mindset: Felt like struggles you have faced will help you grow and change for the better	8.4	(7.3, 8.6)	8.1	(7.5, 8.5)	0.30
Health: Felt physically healthy	7.1	(6.0, 7.4)	7.1	(6.6, 7.9)	0.90
Health: Felt emotionally healthy	6.7	(5.5, 7.0)	6.8	(6.2, 7.3)	0.80
Imagination: Created or thought about something that most would consider unique	7.0	(5.9, 7.3)	6.6	(6.0, 7.3)	0.40
How would you rate your academic ability?	8.5	(7.7, 8.8)	8.4	(8.2, 8.7)	0.60
How would you rate your athletic ability?	7.2	(6.3, 7.6)	7.2	(6.7, 7.7)	0.90
How would you rate your creative ability (music, fine arts, dancing, etc.)?	6.8	(5.7, 7.1)	6.9	(6.3, 7.5)	0.70
Rate your overall wellness	7.9	(7.1, 8.1)	7.4	(7.0, 7.9)	0.10

## 4 DISCUSSION

Results show declines from prior to the COVID‐19 outbreak to 1 month into the pandemic in students' self‐reported levels of wellness in three key areas: maintaining balance in daily life, learning within and outside of school, and spending quality time with friends.

Initial and subsequent overall wellness scores varied across grade level, race/ethnicity, education, and employment status. Although the sample size surveyed was limited, these results provided very early insight into the impact of the pandemic on wellness among a diverse sample of adolescents from one of the largest urban public school districts in the United States. These results align with other early‐pandemic literature showing that factors such as prolonged quarantine, fear of infection, frustration and boredom, lack of in‐person peer contact, shift to virtual learning, lack of personal space at home, and family financial loss have enduring effects on adolescents (Brooks et al., 2020; Lee, 2020; Wang et al., 2020; Zhang et al., 2020).

Home confinement contributes to lifestyle changes and psychosocial stress that can generate and/or worsen child physical and mental health problems (Wang et al., 2020). Throughout child development, but particularly in adolescence, social interaction with peers plays a significant role in wellness. The necessary reduction of social interaction, secondary to physical distancing, remote learning, and sheltering in place, is likely to have contributed to the current mental health crisis among this generation of adolescents.

Adolescent medicine specialists, paediatricians, and behavioural health providers stand in a unique position to consider these effects on adolescent wellness, and to provide a range of support for parents and their teens. The early phase of the COVID‐19 pandemic and the swift lifestyle and educational changes that took place appear to be associated with declines in key areas of adolescent self‐reports of wellness. Additionally, chronic stressors and unexpected losses associated with an ongoing pandemic add complexity to this situation. This underscores the need for more research on adolescent wellness, to identify potentially predictive factors early for clinicians tracking. Adolescents continue to face intermittent remote learning, physical distancing, and limited social interactions, suggesting that wellness information and social support will continue to be needed. In addition to traditional behavioural health care, it is critical that new and accessible behavioural health support (i.e., telehealth and web‐based platforms that provide opportunities for social connection among adolescents) is developed for adolescents.

## CONFLICT OF INTEREST

Dr. Gupta receives research support from the National Institutes of Health (NIH) (R21 ID # AI135705, R01 ID # AI130348, U01 ID # AI138907), Food Allergy Research & Education (FARE), Melchiorre Family Foundation, Sunshine Charitable Foundation, The Walder Foundation, UnitedHealth Group, Thermo Fisher Scientific, and Genentech. She serves as a medical consultant/advisor for Genentech, Novartis, Aimmune LLC, Allergenis LLC, and Food Allergy Research & Education (FARE). She is currently employed by Ann & Robert H. Lurie Children's Hospital of Chicago and is a Professor of Pediatrics & Medicine at Northwestern University Feinberg School of Medicine. The other authors have no conflicts of interest to disclose.

## AUTHOR CONTRIBUTION

Dr. Ogochukwu Ezeoke conceived and designed the survey, drafted the survey tool, assisted in distributing the surveys, analysed and interpreted the data, drafted, and revised the manuscript, and approved the final version as submitted. Madeleine K. Kanaley assisted with the survey development, data analysis, drafted and revised the manuscript, and approved the final version as submitted. Dannielle A. Brown assisted with the survey development, distributing the surveys, and drafted and revised the manuscript, and approved the final version as submitted. Olivia R. Negris assisted with the survey development, distributing the surveys, and drafted and revised the manuscript, and approved the final version as submitted. Rajeshree Das assisted with the survey development, distributing the surveys, and drafted and revised the manuscript, and approved the final version as submitted. Dr. Lisa S. Lombard assisted with the survey development, and drafted and revised the manuscript, and approved the final version as submitted. Dr. Ruchi S. Gupta, MD, supervised the design of the study, drafted and revised the survey, analysed and interpreted the data, drafted and revised the manuscript, and approved the final version as submitted.

**Abbreviation**
WHO: World Health Organization

## Data Availability

The data that support the findings of this study are available from the corresponding author upon reasonable request.
